# Validation of the JEN frailty index in the National Long-Term Care Survey community population: identifying functionally impaired older adults from claims data

**DOI:** 10.1186/s12913-018-3689-2

**Published:** 2018-11-29

**Authors:** Bruce Kinosian, Darryl Wieland, Xiliang Gu, Eric Stallard, Ciaran S. Phibbs, Orna Intrator

**Affiliations:** 1 Center for Health Equity Research and Promotion, Cpl Michael J Crescenz VA Medical Center, Philadelphia, USA; 2 Geriatrics and Extended Care Data Analysis Center, Cpl. Michael J Crescenz VA Medical Center, Philadelphia, USA; 3 Department of Medicine, University of Pennsylvania, Philadelphia, USA; 4 Biodemography of Aging Research Unit, Center for Population Health and Aging, Duke University, Durham, NC USA; 5 Geriatric Research, Education and Clinical Center, VA Medical Center, Durham, NC USA; 6 Health Economics Resource Center, Palo Alto VA Health Care System, Palo Alto, CA USA; 7 Center for Innovation to Implementation, Stanford University School of Medicine, Palo Alto, CA USA; 8 Geriatrics and Extended Care Data and Analysis Center, Palo Alto VA Health Care System, Palo Alto, CA USA; 9 Geriatrics and Extended Care Data and Analysis Center, Canandaigua VA Medical Center, Canandaigua, NY USA; 10 Department of Public Health Sciences, University of Rochester, Rochester, NY USA

**Keywords:** Health services, Functional performance, Deficit accumulation, Community-based, Long term care, Population health management, Long-term institutionalization, Mortality, Predictive validity

## Abstract

**Background:**

Use of a claims-based index to identify persons with physical function impairment and at risk for long-term institutionalization would facilitate population health and comparative effectiveness research. The JEN Frailty Index [JFI] is comprised of diagnosis domains representing impairments and multimorbid clusters with high long-term institutionalization [LTI] risk. We test the index’s discrimination of activities-of-daily-living [ADL] dependency and 1-year LTI and mortality in a nationally representative sample of over 12,000 Medicare beneficiaries, and compare long-term community survival stratified by ADL and JFI.

**Methods:**

2004 U.S. National Long-Term Care Survey data were linked to Medicare, Minimum Data Set, Veterans Health Administration files and vital statistics. ADL dependencies, JFI score, age and sex were measured at baseline survey. ADL and JFI groups were cross-tabulated generating likelihood ratios and classification statistics. Logistic regression compared discrimination (areas under receiver operating characteristic curves), multivariable calibration and accuracy of the JFI and, separately, ADLs, in predicting 1-year outcomes. Hall-Wellner bands facilitated contrasts of JFI- and ADL-stratified 5-year community survival.

**Results:**

Likelihood ratios rose evenly across JFI risk categories. Areas under the curves of functional dependency at ≥3 and ≥ 2 for JFI, age and sex models were 0.807 [95% c.i.: 0.795, 0.819] and 0.812 [0.801, 0.822], respectively. The area under the LTI curve for JFI and age (0.781 [0.747, 0.815]) discriminated less well than the ADL-based model (0.829 [0.799, 0.860]). Community survival separated by JFI strata was comparable to ADL strata.

**Conclusions:**

The JEN Frailty Index with demographic covariates is a valid claims-based measure of concurrent activities-of-daily-living impairments and future long-term institutionalization risk in older populations lacking functional information.

**Electronic supplementary material:**

The online version of this article (10.1186/s12913-018-3689-2) contains supplementary material, which is available to authorized users.

## Background

Recent focus on high value health care—characterized by shifting payment towards desired clinical outcomes—has highlighted the need to account for population differences, such as functional dependency, that can influence those outcomes, but are beyond current risk adjustment models. Frailty--a clinical syndrome characterized by decreased resilience to stressors resulting from dysregulation across multiple physiological systems--increases in prevalence with older age and in women [1] and is associated with a wide range of adverse outcomes. Frailty underlies much old-age disability (e.g., difficulty in performing activities of daily living [ADLs]), and predicts worsening function as well as events such as falls, fractures, intensification of services (e.g., hospital care, and long-term services and supports [LTSS]), and death [2].

Identification of frailty or frailty-related risk subgroups in non-institutionalized older populations has usually required more than demographics and diagnoses routinely collected in electronic health records [EHRs] or available in claims files; it has required information generally undertaken as part of geriatric assessment processes derived from questionnaires, screening, and direct clinical assessment focused on multiple morbidities, specific impairments and disabilities [2–5]. Availability and accessibility of the latter are dependent on the standardization, reach and depth of such assessments in older patient populations as well as the information technology environments. As programs focus resources on high need, high risk elders, the higher rates of frailty in the targeted populations pose a challenge for fairly determining value. For example, in the case of PACE (Program for All-inclusive Care of the Elderly), the Centers for Medicare and Medicaid Services [CMS] distributes a survey to enrolled beneficiaries to determine the level of ADL dependency in the enrolled population, which it uses as a surrogate measure of frailty [6]. Even in health systems committed to uncovering multimorbidity, frail health and functional disabilities, practical challenges may limit the availability and quality of records reflecting these risks [7, 8]. Such information may lie buried in scans or text fields in many EHR systems, or patient records themselves may still not be integrated across multiple provider and insurer systems, subjecting their population-level uses to indication and selection biases. In contrast, employing diagnoses to identify elderly subgroups bearing frailty-related risk for poor outcomes would facilitate comparative effectiveness analyses, health planning and management, and pay-for-performance adjustments in populations whose underlying frail health and/or disabilities are mostly unknown or inaccessible.

The JEN Frailty Index [JFI] produces a computational phenotype based on ICD-9/10 diagnostic codes recoverable from U.S. Medicare claims data; it was designed to be highly predictive of long-term institutionalization [LTI], and thus risk of high LTSS expenditures. As a proprietary tool, details of its development have not been published, although the JFI has been employed to control for LTI risk and high LTSS expenditures in studies of U.S. community-care interventions [9–12]. The JFI is calculated over 13 categories of diagnostic codes representing geriatric syndromes, functional deficits and multimorbidity clusters, the accumulation of which is the JFI score. The developers optimized prediction of LTI in a dual-eligible [Medicare and Medicaid] sample, which included both elderly and non-elderly (younger adult) at-risk beneficiaries [13], and have suggested that higher JFI scores predict ADL dependency, providing a method to identify disabled population subgroups where diagnostic data are known, but functional status is not.

We examine the relationship of JFI to concurrent ADLs and incident LTI in the elderly (65+) U.S. population using a dataset linking the National Long-Term Care Survey [NLTCS] to CMS and Veterans Health Administration [VHA] claims and service utilization files. Nearly a quarter of NLTCS community respondents were VHA-enrolled veterans, so merger of CMS and VHA files allowed for a fuller accounting of diagnoses and LTSS utilization in the sample. In validity tests of the JFI—operationalized as concurrent ADL dependency and 1-year LTI risk—we address the following: does the JFI discriminate those with ≥2 or ≥ 3 ADL dependencies at the time of survey; does the JFI discriminate non-institutionalized individuals who will incur LTI over a 12-month period; do JFI- and ADL-based prediction models similarly discriminate those incurring LTI; and, do JFI and ADL risk groups have similar long-term community survival?

## Methods

### Population and data sources

The 2004 NLTCS is a survey of U.S. disabled and nondisabled older adults including both institutional and community populations [14]. Our study was limited to the community sample and those in fee-for-service Medicare for the prior year. Demographic and functional status data were obtained from detailed interviews when available, or recovered from the screener (*per* NLTCS protocol, respondents not having basic or instrumental ADL [IADL] dependencies were not interviewed). Survey information was linked to CMS claims, Minimum Data Set [MDS] files, and vital statistics data, and matched to VHA end-of-year enrollment files [15]. Composite CMS-VHA claims data allowed construction of a complete JFI score, relating that score to the individual’s functional status at survey time, and 5-year LTI and survival status, following a ninety-day post-baseline maturation period which allowed for new nursing-home [NH] placements to qualify as LTI.

### Predictor variables

We classified disability status as no impairment, IADL difficulty only, or dependency in each of six ADLs (bathing, continence, dressing, eating, toileting, and transferring), with dependency defined at or above needing personal standby help with or without special equipment. ADL impairment counts are used in modeling LTI, wherein non-impaired subjects and those with only IADL difficulty receive a zero score.

The JFI software program was licensed to VHA by JEN Associates [13]. The algorithm developed index scores from nearly 1800 CMS diagnosis codes recovered from fee-for-service Medicare claims and VHA face-to-face diagnoses in the year prior to interview or screening. The 13 JFI domains are: minor ambulatory limitations, severe ambulatory limitations, chronic mental illness, chronic developmental disability, dementia, sensory disorders, self-care impairment, syncope, cancer, chronic medical disease, pneumonia, renal disorders, and other systemic disorders. The JFI score is the unweighted sum of the condition domains triggered. Scores can be treated as a linear categorical variable or be grouped into risk strata. We report the JFI mean, risk stratum distributions, and domains triggered (Table 1). JFI score counts are used in all models.

**Table 1:** Sample Characteristics for ADL Identification and Death/LTI Prediction Analyses^1^

2004 NLTCS Non-HMO Community Sample at Baseline	*A: ADL Analysis* *n* = 12,752	*B: LTI Analysis* *n* = 12,563
	count (%) or mean	count (%) or mean
*Sociodemographics*		
Age	77.3	77.1
Male	5317 (41.7)	5242 (41.7)
Married	6673 (52.3)	6614 (52.6)
Race/ethnicity		
White	11,102 (87.1)	10,935 (87.0)
African-American	832 (6.5)	820 (6.5)
Hispanic	543 (4.3)	536 (4.3)
Education		
≤9th Grade	2230 (17.4)	2177 (17.3)
10th–12th Grade	5282 (41.4)	5210 (41.5)
Some college	2793 (21.9)	2763 (22.0)
≥College graduate	2093 (16.4)	2075 (16.5)
Has Identified Family Caregiver	1645 (12.9)	1565 (12.5)
Enrolled in VHA System	3424 (26.9)	3374 (26.9)
Used VHA System in Prior Year	894 (7.0)	878 (7.0)
Medicaid Enrolled	1581 (12.4)	1504 (12.0)
U.S. Region		
Northeast	2474 (19.4)	2437 (19.4)
North Central	3405 (26.7)	3357 (26.7)
South	4667 (36.6)	4599 (36.6)
West	2193 (17.2)	2170 (17.3)
*Selected NLTCS Chronic Conditions*		
Osteoarthritis and Rheumatoid Arthritis	6398 (50.2)	6283 (50.0)
Hyperlipidemia	5081 (39.9)	5038 (40.1)
Hypertension	7522 (59.0)	7390 (58.8)
Ischemic Heart Disease	4493 (35.2)	4375 (34.8)
Diabetes	2431 (19.1)	2375 (18.9)
Chronic Obstructive Pulmonary Disease	1648 (12.9)	1590 (12.7)
*Disability*		
Non-Disabled	9990 (78.3)	9929 (79.0)
IADL Difficulty Only	412 (3.2)	410 (3.3)
1–2 ADL Dependencies	1074 (8.4)	1044 (8.3)
3–4 ADL Dependencies	664 (5.2)	644 (5.1)
5–6 ADL Dependencies	612 (4.8)	536 (4.3)
*JEN Frailty Index*	2.05	2.02
Low Frailty (score 0–3)	10,349 (81.2)	10,265 (81.7)
Moderate Frailty (4–5)	1715 (13.5)	1667 (13.3)
High Frailty (6–7)	588 (4.6)	541 (4.3)
Very High Frailty (≥8)	100 (0.8)	90 (0.7)
*JFI Domains (prevalence)*		
Minor ambulatory limitations	7498 (58.9)	7354 (58.5)
Severe ambulatory limitations	1196 (9.4)	1155 (9.2)
Chronic mental illness	1294 (10.2)	1250 (10.0)
Chronic developmental disability	5 (0.04)	5 (0.04)
Dementia	534 (4.2)	490 (3.9)
Sensory limitations	1001 (7.9)	982 (7.9)
Self-care impairment	2199 (17.2)	2114 (16.8)
Syncope	2279 (17.9)	2221 (17.7)
Cancer	1286 (10.1)	1249 (9.4)
Chronic medical disease	6003 (47.1)	5874 (46.8)
Pneumonia	497 (3.9)	462 (3.7)
Renal disorders	338 (2.7)	315 (2.5)
Other systemic disorders	2004 (15.9)	1921 (15.3)

Age and gender taken from the survey were screened as outcome predictors using bivariate tests and evaluated for inclusion in the multivariable models.

### Outcome measures

ADL impairment as a binary dependent variable was assigned threshold values at ≥2 and ≥ 3. We followed prior work in identifying LTI using MDS records [16]. LTI outcome was determined for all respondents. Generally, the “LTI flag” was raised on the date of the first quarterly MDS assessment following a dated admission assessment, indicating 90-days of NH residence, although variable timing of quarterly assessments for some led to reassignment of their LTI dates to the 90-day mark, accounting for other service history. For VHA users, 90-day cumulative VHA NH residence could also trigger LTI. Information on VHA LTI was obtained from the Geriatrics and Extended Care [GEC] residential history file developed by the GEC Data and Analysis Center. For LTI, we excluded individuals whose admission MDS assessments predated their NLTCS interviews or who had quarterly assessments in the first follow-up quarter. Because of the exclusion of prevalent NH cases in LTI analyses and the requirement for a 90-day stay to trigger LTI, the observation period extended from the beginning of the second quarter through the fifth quarter of follow-up to define a full at-risk year. Finally, we tracked mortality from index date through the third quarter of the fifth follow-up year. This identified deaths occurring prior to any LTI as an alternative response level in multinomial logistic regression analyses of one-year (i.e., Q2-Q5) outcomes [17], and allowed construction of 5-year “community survival” curves (i.e., survival net of death and LTI) for contrasting performance of JFI and ADL risk strata.

### Statistical methods

Analysis addressed two properties of a prognostic index: calibration and discrimination [18]. Calibration requires that the risk for a predicted group is close to the observed risk for its individuals, and—in this context--that as the predicted risks rise with higher JFI scores, the risk for ADL dependency and LTI rise. The JFI was partitioned into LTI-risk groups, for which we constructed likelihood ratios [LRs] for ADL impairment and LTI, representing the true positive rate (i.e., sensitivity) of JFI for the group (JFI score range), divided by the group’s false positive rate (i.e., 1- specificity). Calibration was further tested in multivariate analyses by dividing the population into JFI deciles based on the predicted risk of ADL dependency and LTI, then comparing observed to predicted risk within deciles using the Hosmer-Lemeshow [H-L] χ^2^ test [19].

Discrimination is the ability to separate a population on having a condition or experiencing an event. Binomial logistic regressions tested whether JFI discriminated individuals having multiple ADL dependencies (i.e., ≥ 2 or ≥ 3). Multinomial logistic regression was used to test whether JFI and ADLs discriminate individuals who incurred LTI in the 1-year risk period, net of prior death, comparing the ability of the covariate-adjusted models to discriminate incident LTI. Both sets of analyses produced areas under receiver operating characteristic curves (AUCs) as discrimination indicators [20]. AUC contrast tests weigh the impact on AUC of adding index risk scores (JFI for ADL dependency identification, and JFI and ADL count for LTI) to demographic predictors (age and/or sex) [21, 22]. To assess overall accuracy, Brier scores and pseudo-R^2^ values were calculated [18]. Finally, we constructed two stratified sets of 5-year Kaplan-Meier curves with 95% Hall-Wellner bands to assess community survival based on ADL and JFI risk.

SAS version 9.4 software was used to perform univariate, bivariate and standard rate procedures for descriptive statistics, and logistic regression and multinomial logistic regression for concurrent identification and prediction modeling. Analysis did not employ NLTCS survey weights as our objective was validation of the JFI and not estimation of population rates.

## Results

The 2004 NLTCS was comprised of 20,474 persons [23]. Excluding the institutional sample and subjects with prior-year HMO enrollment reduced the sample (12,752) used for JFI identification of ADL dependency (Table 1, Column A). This sample was further reduced to 12,563 for LTI prediction by excluding 50 individuals in NHs on their screening/interview dates, and 139 not surviving the maturation quarter (Table 1, Column B). The mean age in both cohorts was about 77 years; 42% were males, and 87% Caucasian.

### Identification of ADL dependency

Ten percent (1276) of the full community sample (12,752) were impaired in three or more ADLs, and 13.7% (1752) were impaired at ≥2 ADLs (Table 2). The sample was cross-classified by JFI-score risk categories: low (0–3), moderate (4–5), high (6–7) and very high risk (≥8) and separately by ADL impairment groups. Most subjects (ADL impaired and relatively independent) had low JFI risk, with decreasing numbers in successively higher risk strata (Table 1, Table 2). The likelihood ratios [LRs] at both ADL thresholds show a strong relationship between higher JFI scores and ADL impairment. The LR gradient for the ≥3 ADL threshold ranges from 0.67 to 10.56; the ≥2 ADL gradient was steeper (0.69–11.06). For both, classifications were highly specific, with good positive predictive values [PPVs]--individuals identified by high JFI scores are very likely to have dependency: e.g., of the 4% with JFI scores 8+, 64% have ≥2 ADL impairments (Table 2).

**Table 2:** Concurrent Activities of Daily Living [ADLs] at ≥2 and ≥ 3 Dependencies by JEN Frailty Index [JFI] Risk, 2004 NLTCS Community Sample (*n* = 12,752)^2^

ADL Groupings	JFI 0–3	JFI 4–5	JFI 6–7	JFI ≥8	JEN Frailty Index ≥6			JEN Frailty Index ≥8		
	(row percentages)				Sens	Spec	P/NPV	Sens	Spec	P/NPV
0–2 (*n* = 11,476)	9633 (83.9)	1396 (12.2)	401 (3.5)	46 (0.4)	18.9% (16.8, 21.2)	96.1% (95.7–96.4)	35.0%/91.4%	4.2% (3.2, 5.5)	99.6% (99.5, 99.7)	54.0%/ 90.3%
3–6 (*n* = 1276)	716 (56.1)	319 (25.0)	187 (14.7)	54 (4.2)						
***Likelihood Ratios***	***0.67***	***2.06***	***4.19***	***10.56***						
0–1 (n = 11,000)	9328 (84.8)	1292 (11.8)	344 (3.1)	36 (0.3)	17.6% (15.8, 19.5)	96.5% (96.1, 96.8)	44.8%/ 88.0%	3.7% (2.8, 4.7)	99.7% (99.5, 99.8)	64.0%/ 86.7%
≥2 (*n* = 1752)	1021 (58.3)	423 (24.1)	244 (13.9)	64 (3.7)						
***Likelihood Ratios***	***0.69***	***2.05***	***4.45***	***11.06***						

Bold data are important result

In multivariate binomial logistic regression analyses, the odds ratios of the JFI score were approximately 1.4 (*p* < 0.001) in both ADL threshold models--an increase of over 40% in risk of concurrent impairment *per* JFI unit increase (Table 3). Higher age and female sex are also predictive: each added year increases the impairment odds 10–11%; while females have about a one-third greater risk of impairment at either threshold. AUCs for both models indicate very good discrimination, at 0.807 for ≥3 ADL threshold, and 0.812 for the ≥2 ADL threshold. H-L tests indicate good fit to the data (see Additional file 1: Figure S1A). The Brier scores indicate very good overall model performance (scores < 0.1), as do the pseudo-R^2^s—at 0.24 and 0.27. Using the final three-factor ADL identification model at the ≥2 ADL threshold as reference (AUC = 0.812), the age-only AUC was 0.756 (contrast χ^2^*, p* < 0.001), and the age + JFI model AUC equaled 0.807 (*p* = 0.001), indicating that the three-factor identification model has superior discrimination (Additional file 1: Figure S1B).

**Table 3:** JEN Frailty Index Identification of Concurrent ADL Dependencies with Gender and Age Controls, 2004 NLTCS Community Sample (*n* = 12,752)^3^

*Multivariable Models* Predictors	Log Odds [Wald 95% c.i.]	Area under ROC Curve [95% c.i.]	Hosmer-Lemeshow χ^2^ (*p*)	Brier Score [pseudo-R^2^]
*≥3 ADL Dependencies* (n = 1276)				
Age	1.105 [1.097, 1.113]	0.807 [0.795, 0.820]	5.110 [d.f. = 8] (0.746)	0.076 [0.240]
Gender	0.632 [0.550, 0.725]			
JFI Score	1.403 [1.359, 1.448]			
*≥2 ADL Dependencies* (n = 1752)				
Age	1.110 [1.103, 1.118]	0.812 [0.801, 0.822]	5.997 [d.f. = 8] (0.648)	0.096 [0.274]
Gender	0.633 [0.561, 0.715]			
JFI Score	1.423 [1.382, 1.464]			

### JFI v. ADL prediction of mortality and LTI in the one-year event window

LTI incidence was low (156 events). In contrast, there were 605 deaths during the same period (Q2-Q5 *post* screening/interview), in addition to 139 deaths in the post-index 90-day, pre-LTI observation interval). By the end of follow-up, there were 2954 deaths and 755 LTI events, or about 4 deaths *per* LTI event.

LTI risk rose evenly from 0.9 to 4.4% (lowest to highest risk JFI categories), and from 0.6 to 6.7% in the corresponding ADL categories (Table 4). Only 17.3% of all LTI cases fell into the high and very high JFI risk categories, whereas at and above the corresponding ADL threshold (≥ 3 impairments) 45.5% of LTI cases were captured. The LR gradients for the JFI and ADL risk groups are 0.75–3.71 and 0.45–5.78, respectively. Setting JFI thresholds at ≥6 and ≥ 8 showed both to be highly specific (> 95%), although PPVs are low (< 5%). Similarly, at ADL thresholds of ≥3 and ≥ 5, the ADL predictions were also specific (91.1, 96%), with low PPVs (6, 6.7%). Because of the high specificity of JFI (95.1, 99.3% in the high categories), the likelihood ratios are similar for LTI across comparable ADL-count and JFI groups.

**Table 4:** One-Year Long-Term Institutionalization [LTI] at Different Thresholds by JFI and ADL Groupings, 2004 NLTCS Community Sample (n = 12,563)^4^

Outcome	JFI 0–3	JFI 4–5	JFI 6–7	JFI ≥8	JEN Frailty Index ≥6			JEN Frailty Index ≥8		
	(row percentages)				Sens	Spec	P/NPV	Sens	Spec	P/NPV
No LTI (n = 12,407)	10,169 (82.0)	1634 (13.2)	518 (4.2)	86 (0.7)	17.3% (11.9, 24.4)	95.1% (94.7–95.5)	4.3%/ 98.9%	2.6% (0.8, 6.8)	99.3% (99.1, 99.4)	4.4%/ 98.8%
LTI (*n* = 156)	96 (61.5)	33 (21.2)	23 (14.7)	4 (2.6)						
*Likelihood Ratios*	***0.75***	***1.61***	***3.50***	***3.71***						
Outcome	ADL = 0	ADL 1–2	ADL 3–4	ADL 5–6	ADL Dependencies ≥3			ADL Dependencies ≥5		
No LTI (n = 12,407)	10,281 (82.9)	1017 (8.2)	609 (4.9)	500 (4.0)	45.5% (37.6, 53.7)	91.1% (90.5, 91.6)	6.0%/ 99.3%	23.1% (16.9, 30.6)	96.0% (95.6, 96.3)	6.7%/ 99.0%
LTI (n = 156)	58 (37.2)	27 (17.3)	35 (22.4)	36 (23.1)						
*Likelihood Ratios*	***0.45***	***2.11***	***4.57***	***5.78***						

Bold data are important result

Two multinomial logistic regression analyses sorted on mortality and LTI outcomes (Table 5). For mortality, the AUC for JFI with demographic covariates was 0.76 [95% c.i.: 0.74, 0.78], with good calibration (H-L χ^2^, *p* = 0.350) and pseudo-R^2^ (0.126); older subjects were at risk, male sex almost doubled the mortality risk, and the odds ratio for JFI was highly significant at 1.18--an 18% increase in mortality risk *per* JFI unit. For LTI, the multivariable AUC was higher (0.78 [0.75, 0.82]) with greater calibration and a lower Brier score indicating very good predictive accuracy (see Additional file 1: Figure S2A); again, increasing age was a significant risk, but the gender risk was not significant. JFI increase was predictive (OR = 1.25), raising LTI risk by 25%. The AUC for JFI alone was only fair (0.65), *v.* the AUC for age (0.76) (Additional file 1: Figure S2B). Age and JFI in combination significantly increased the LTI AUC (*p* = 0.015) compared to age alone.

**Table 5:** Multinomial Prediction of LTI and Death without Prior LTI in Q2-Q5 (1 Year), 2004 NLTCS Community Sample (*n* = 12,563). (“Neither event” is reference category)

*Multinomial Models* Predictors	Log Odds [Wald 95% c.i.]	Area under ROC Curve [95% c.i.]	Hosmer-Lemeshow χ^2^ (*p*)	Brier Score [pseudo-R^2^]
*Multivariable Model with JFI* Response = Death (*n* = 605)				
Age	1.090 [1.080, 1.100]	0.759 [0.074, 0.778]	8.906 [d.f. = 8] (0.350)	0.044 [0.126]
Gender	1.983 [1.657, 2.372]			
JFI Score	1.178 [1.129, 1.230]			
Response = LTI (n = 156)				
Age	1.096 [1.076, 1.115]	0.781 [0.747, 0.815]	6.956 [d.f. = 8] (0.542)	0.012 [0.102]
Gender	0.780 [0.556, 1.122]			
JFI Score	1.249 [1.157, 1.348]			
*Multivariable Model with ADL* Response = Death (n = 605)				
Age	1.070 [1.059, 1.080]	0.768 [0.748, 0.788]	13.747 (0.089)	0.042 [0.151]
Gender	2.098 [1.755, 2.508]			
ADL Count	1.395 [1.338, 1.455]			
Response = LTI (n = 156)				
Age	1.068 [1.049, 1.088]	0.829 [0.799, 0.860]	41.233 (< 0.001)	0.012 [0.125]
Gender	0.962 [0.673, 1.375]			
ADL Count	1.391 [1.291, 1.499]			

Turning to the ADL multinomial models with covariates, mortality discrimination was very good and slightly better than the JFI-based model (AUC = 0.77 [95% c.i.: 0.75, 0.79]), although marginally calibrated (H-L χ^2^, *p* = 0.09). As in the JFI-based model, both covariates predicted death, with comparable effects: a 7% *per* year of age risk increment, and a doubling of male mortality risk. Each additional ADL dependency raises mortality risk by 40% (equivalent to JFI incremental risk after accounting for scaling factors). Discrimination of the ADL-based LTI model was also similar and somewhat better than the JFI-based model (AUC = 0.83 [0.80, 0.86]).

### Long-term community survival

**Fig. 1:**
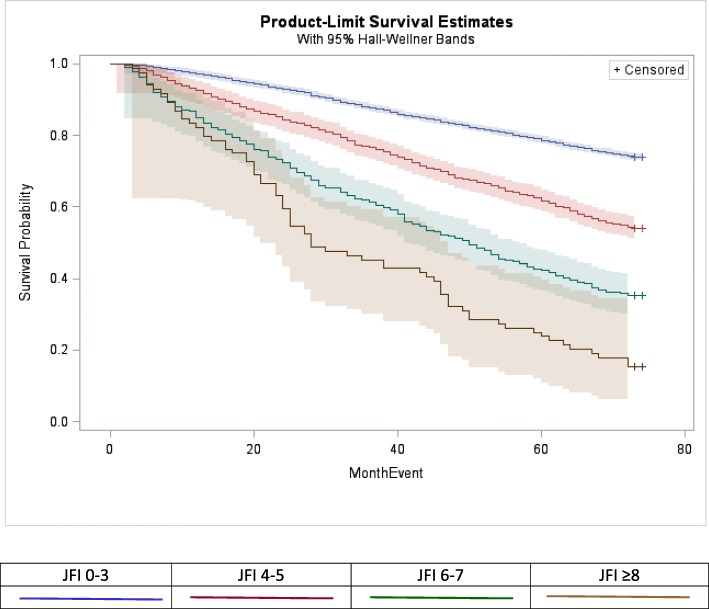
Product-Limit Estimates of Five-Year Community Survival by JFI Risk Categories, NLTCS 2004 Community Sample (n = 12,702)^5^

**Fig. 2:**
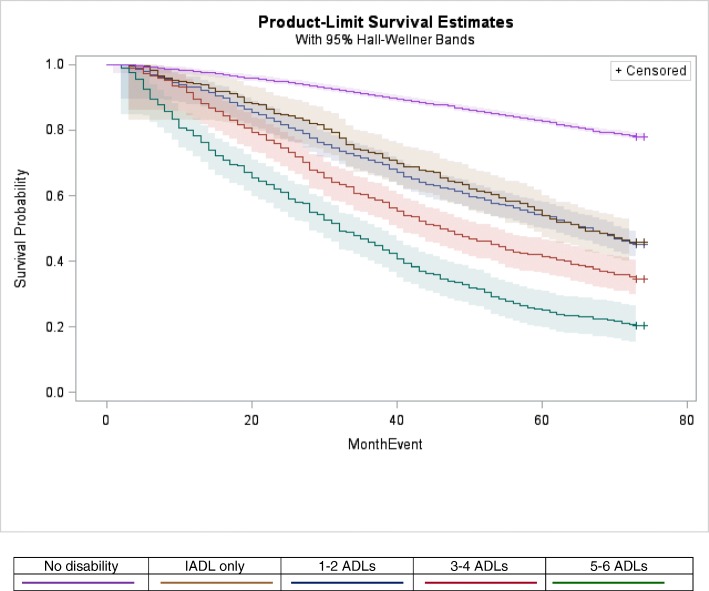
Product-Limit Estimates of Five-Year Community Survival by Disability Risk Groups, NLTCS 2004 Community Sample (*n* = 12,702)

The comparability of JFI and ADL risk for both long-term death and LTI was illustrated by 5-year community survival curves (Figs. 1 and 2). Both ADL and JFI risk strata follow divergent trajectories across community survival space, with two exceptions: while the very high frailty curve (JFI ≥ 8) dropped well below the high-risk curve (JFI 6–7), their 95% bands overlapped, due to band breadth of the sparse, very-high risk curve; and the IADL-only impaired and 1–2 ADL impairments follow similar trajectories. Community survival of the moderate-risk stratum (JFI 4–5) tracks closely with the IADL only/1–2 ADL impairment curves; and high risk (JFI 6–7) track close to the 3–4 ADL curve.

## Discussion

The JEN Frailty Index identified ADL impairment and predicted LTI in a representative older U.S. community population. In comparing the test performance of ADLs and JFI for LTI (Table 4), JFI has excellent specificity, but was less sensitive than ADLs, implying that--while JFI is useful for identifying comparably at-risk individuals in program evaluations due to its high specificity, and identifying populations for targeting services--individual assessment is still essential for service deployment. The JFI discriminates well at two commonly used ADL thresholds for service targeting, and good discrimination of LTI risk (approaching that of an ADL-based model) with the addition of an age covariate: age increases the JFI’s AUC by 20% (from 0.65 to 0.78). JFI’s developers did not find age important in targeting JFI to LTI in the Medicare-Medicaid population, which included developmentally disabled and medically fragile younger adults (age > 18). JFI’s inclusion of chronic developmental disability signals this difference (its prevalence in our NLTCS sample is very small). Long-term community survival--an important emerging quality metric [24]--was similar by JFI or ADL risk level. This is promising for comparative effectiveness studies tracking longitudinal outcomes: while current claims-based studies use ADL assessments from utilization-based tools (such as MDS and OASIS), access is highly selected (one needs, respectively, a NH stay or an episode of home health care) and assessment timings are highly variable in relation to the period of program exposure. JFI provides a way to align for functional dependency and LTI risk at the inception of an index event.

Very recently, others have also developed and validated EHR- and claims-based frailty and geriatric-risk indices [8, 25–30]. These were not catalogued in an excellent review of frailty instruments [4], nor in a review of earlier efforts to measure frailty using claims provided by Kim and Schneeweiss [31]. Two European groups developed and validated frailty indices constructed on a deficit accumulation template consistent with recommendations of Searle et al. [32], taking advantage of advances in those countries in creating large primary-care records registries which also integrate patient records across relevant data fields [25, 26]; in addition to these indices being useful for records-based risk screening, they may hold value for research on the biomarkers, etiology, and sequelae of frailty however it may be defined [7]. Three of four [27–30] American efforts are based solely on Medicare claims, reflective perhaps of the immature state of long-promised EHR integration in the U.S., but which take advantage of the position of Medicare as a near universal payer of health services (across sectors and providers) for American elders. Two [27, 28] make Fried’s physical frailty phenotype [33] the focus of content development and—in one case—the chief validation target [28]. In contrast, the claims-based instrument of Kim et al. [30]—which like the JFI was constructed on a deficit accumulation model--explicitly employed a survey-based frailty index as a concurrent development target. The work of Kan et al. [8] altogether eschews frailty constructs, templates and targets in providing a “geriatric risk” index; it is the sole American effort to go beyond claims files, adding EHR data from structured tables, text fields and scans (demonstrating incremental prediction improvements with the addition of these data sources). Finally, both Faurot’s frailty-related measure [27] and our JFI validation focused on ADL disability (at different dependency thresholds) for concurrent prediction, both demonstrating very good discrimination.

While each of these new measures demonstrates discrimination on a variety of outcomes, the JFI—to date uniquely—is a particular predictor of long-term institutionalization (it has been employed to control for high LTSS expenditure risk [9–12]). This is not equivalent to predicting all NH admissions (as several of these indices demonstrate), which include various kinds of short stays (respite use, post-acute care). Future JFI development will need to consider recalibration for current LTSS use profiles and expenditures, given the “rebalancing” of LTSS away from institutions and towards higher intensity community-based services which may alter the relationship between LTI and high LTSS expenditure. In addition, outcomes such as community survival and other disability- and frailty-related endpoints should be studied, and--where appropriate—compared to predictions obtained from alternative measures.

## Conclusion

The JFI is a valid measure of risk for concurrent ADL dependency and incident long-term institutionalization in studies of older populations covered by Medicare or otherwise described by ICD-9/10 diagnosis codes. It should perform well as a surrogate for ADLs in matching patients for comparative effectiveness research, screening of subjects for inclusion or exclusion in research, grading of population risk, and other purposes. The JFI may capture elements of frail health not registered by ADLs, but this remains to be evaluated. For individual risk assessment and service planning, the JFI does not substitute for frailty or ADL assessments, and related clinical evaluations. But when combined with age and gender, JFI provides a means to predict mortality and LTI in the absence of unbiased assessments of functional disabilities.

## Additional files

Additional file 1: **Figure S1A.** Calibration Plot for JFI + Age + Gender Model Identifying Subjects with ≥2 Concurrent ADL Dependencies. **B**: ROC Curve Contrasts for the ≥2 ADL Dependency Models (age; age + JFI; age + JFI + gender). **Figure S2A.** Calibration Plot for JFI + Age Model Predicting Long-Term Institutionalization in the One-Year (Q2-Q5) Follow-Up Window. **B**: ROC Curve Contrasts for Long-Term Institutionalization in the One-Year Follow-Up Window. (DOCX 116 kb)

## Abbreviations

ADL: Activities of daily living
AUC: Area under a receiver operating characteristic curve
CCI: Charlson comorbidity index
CFI: Claims-based frailty indicator
CMS: Centers for Medicare and Medicaid Services
CPT: Current Procedural Terminology
GEC: VHA Geriatrics and Extended Care
HCPCS: Healthcare Common Procedure Coding System
H-L: Hosmer-Lemeshow test statistic
HMO: Health Maintenance Organization
HR: Hazard ratio
IADL: Instrumental activities of daily living
ICD-9: International Classification of Diseases, 9th edition
JFI: JEN Frailty Index (“JEN” is not an acronym but a concept reflecting Confucian virtues of protection and altruism)
LR: Likelihood Ratio
LTI: Long-term institutionalization
LTSS: Long-term services and supports
MCBS: Medicare Current Beneficiaries Survey
MDS: Minimum Data Set
NH: Nursing home
NLTCS: National Long-Term Care Survey
NPV: Negative predictive value
OASIS: Outcome and assessment Information Set
OR: Odds ratio
PPV: Positive predictive value
Q: Quarter-year
ROC: Receiver operating characteristic
SD: Standard deviation
Sens: Sensitivity
Spec: Specificity
VHA: Veterans Health Administration

## Footnotes

1 Difference due to 198 exclusions of prevalent NH/LTI cases at baseline interview and deaths in the first quarter of follow-up.

2 Sensitivity (sens) and specificity (spec) as percentages with 95% confidence intervals; P/NPV = positive and negative predictive values.

3 The AUC indicates the discrimination of the prediction model; the Hosmer-Lemeshow χ^2^ is a measure of the fit of data to the model, or calibration (higher *p*-values of the statistic indicating better calibration); the Brier score and pseudo-R^2^ assess overall performance (Brier scores range from 0 to 1, lower scores indicating better performance).

4 Excludes prevalent LTI cases and deaths.

5 Cohort denominator includes persons who died in first quarter. All subjects were followed through the third quarter of the fifth year.
