# Supplementary material for: Validation of the JEN frailty index in the National Long-Term Care Survey community population: identifying functionally impaired older adults from claims data
Source: BMC Health Serv Res. 2018 Nov 29;18:908. doi: 10.1186/s12913-018-3689-2 (PMC6267903; doi:10.1186/s12913-018-3689-2)
Supplement: Supplementary file 1 — Figure S1A. Calibration Plot for JFI + Age + Gender Model Identifying Subjects with ≥2 Concurrent ADL Dependencies. B: ROC Curve Contrasts for the ≥2 ADL Dependency Models (age; age + JFI; age + JFI + gender). Figure S2A. Calibration Plot for JFI + Age Model Predicting Long-Term Institutionalization in the One-Year (Q2-Q5) Follow-Up Window. B: ROC Curve Contrasts for Long-Term Institutionalization in the One-Year Follow-Up Window. (DOCX 116 kb) [file 12913_2018_3689_MOESM1_ESM.docx]

Figure S1A: Calibration Plot for JFI+Age+Gender Model Identifying Subjects with ≥ 2 Concurrent ADL Dependencies

Figure S1B: ROC Curve Contrasts for the ≥ 2 ADL Dependency Models (age; age + JFI; age + JFI +gender)

Figure S2A: Calibration Plot for JFI+Age Model Predicting Long-Term Institutionalization in the One-Year (Q2-Q5) Follow-Up Window^[[1]](#footnote-1)^

Figure S2B: ROC Curve Contrasts for Long-Term Institutionalization in the One-Year Follow-Up Window

1. *Per* Table 5, the Hosmer-Lemeshow χ^2^_(df=8)_ = 6.956; *p*=0.5415, indicative of good calibration, or model fit. [↑](#footnote-ref-1)
